# Usability Testing of a Web Tool for Dissemination and Implementation Science Models

**DOI:** 10.1007/s43477-024-00125-7

**Published:** 2024-06-14

**Authors:** Rebekah Natalie Gomes, Bryan S. Ford, Rachel G. Tabak, Ross C. Brownson, Sara Malone, Maggie Padek, Russell E. Glasgow, Borsika Rabin

**Affiliations:** 1 University of Colorado Anschutz Medical Campus, Aurora, CO USA; 2 Colorado Implementation Science Center for Cancer Control (COISC3), Aurora, CO USA; 3 Brown School, Washington University, St. Louis, MO USA; 4 Prevention Research Center, Brown School at Washington University in St. Louis, St. Louis, MO USA; 5 Department of Surgery, Division of Public Health Sciences, Alvin J. Siteman Cancer Center, Washington University School of Medicine, Washington University in St. Louis, St. Louis, MO USA; 6 Frontiers Clinical and Translation Science Institute, University of Kansas Medical Center, Shawnee Mission Parkway, Fairway, KS USA; 7 Herbert Wertheim School of Public Health and Human Longevity Science, University of California San Diego, La Jolla, CA USA; 8 Dissemination and Implementation Science Center, UC San Diego Altman Clinical and Translational Research Institute, University of California San Diego, La Jolla, CA USA; 9 University of Colorado School of Medicine, Mailstop F443, 1890 North Revere Court, Suite P12-3200, Aurora, CO 80045 USA

**Keywords:** Dissemination, Implementation, Dissemination and implementation models, User testing, Web tools

## Abstract

Dissemination and Implementation science is dedicated to increasing the speed of evidence-based research translated into practice as guided by one or multiple D&I theories, models, and frameworks. The Dissemination and Implementation Models in Health Research and Practice web tool guides users on how to plan, select, combine, adapt, use, and assess theories, models, and frameworks. This paper describes usability testing to update the web tool. Iterative user testing was conducted with implementation science research and clinical participants to facilitate updates and optimize the functionality of the tool. A multi-step protocol involved quantitative and qualitative data collection including a survey, interviews, and a usability testing session. Data from the pre-testing surveys were summarized as frequencies. Data from the usability testing sessions were analyzed using a hybrid adapted deductive rapid matrix qualitative analysis. Data from the interviews were analyzed by deductive a priori coding. Fifteen interviewees represented different research and clinical groups and levels of expertise utilizing D&I TMFs. Participants were purposively selected to represent a range of disciplines and D&I expertise, all invited via one-time email. The 847 total interview comments were reduced by similarity to 259 comments, and 142 were feasible changes fitting the priorities of the web tool. Changes to content, format, and functionality are described in this paper. The iterative usability testing elicited improvements to the web tool including adding more examples, definitions, visuals, and tutorials and simplifying the written content. The web tool remains flexible for additions concerning health equity, de-implementation, and other issues.

**Supplementary Information:**

The online version contains supplementary material available at 10.1007/s43477-024-00125-7.

## Introduction

Dissemination and Implementation (D&I) science is dedicated to increasing the speed and amount of evidence-based research translated into real-world practice. The use of D&I theories, models, and frameworks (TMFs) can make this process more rigorous, systematic, generalizable, and as a result, more impactful especially regarding considerations of equity (Snell-Rood et al., 2021; Tabak et al., 2012). Many D&I TMFs emerged over the past few decades—per the most recent count by Presseau and colleagues as many as 159 (Baumann et al., 2022; Presseau et al., 2022; Strifler et al., 2018). Navigating decisions about which D&I TMFs to use and how to operationalize the TMFs in a study requires guidance and skills (Moullin et al., 2020), and can sometimes seem overwhelming, especially for those who are newer to the field of D&I. Selecting (or adapting) TMFs is one of the most important decisions in D&I projects and yet there is little concrete guidance available (Birken et al., 2017). To our knowledge, there is no guidance available that contains information on the key content of potential TMFs. Even more seasoned D&I researchers can benefit from a systematic summary of existing models and examples of how these can be operationalized. While access to formal D&I training, mentoring, and technical assistance is broadening, these services remain limited to only a small proportion of researchers and practitioners interested in D&I research (Ford et al., 2018; Tabak et al., 2021). Interactive web tools that provide step-by-step guidance on how to integrate D&I science concepts can play a key role in building capacity and support for D&I research including the use of D&I TMFs (Domagk et al., 2010; Ford et al., 2018; Tabak et al., 2021; Trinkley et al., 2023).

The Dissemination and Implementation Models in Health Research and Practice web tool (D&I TMFs web tool) (Ford et al., 2018; Glasgow et al., 2014–2022) is one such resource. It was launched in 2014 to provide an interactive, searchable compilation of the various D&I TMFs as well as guidance on selecting the most relevant TMFs for one’s project. Equally important, the web tool provides guidance for and examples of combining, adapting, using, and assessing D&I TMFs across the life course of a research project. The D&I TMFs web tool is a free, publicly available resource. The first version of the web tool was created with funding from the National Cancer Institute through a primary supplement to the Centers for Excellence in Cancer Communication Research initiative. Since its original launch, various federal grants and institutional sources have supported its general maintenance, expansion, and updating (including the one described in this paper). The web tool was initially populated with the TMFs identified in the reviews by Tabak et al. (2012) and Mitchell et al. (2010). Additional TMFs have been added over the past seven years based on emerging reviews of TMFs and recommendations from D&I experts. As of the writing of this paper, the web tool includes 114 TMFs further cataloged by: focus (dissemination and/or implementation activities), construct flexibility, socio-ecological level, field of origin, and intended user (researcher versus practitioner). Elements of each TMFs are abstracted using a standardized process and paired to a list of constructs allowing for linkage across models. In addition, key citations, citations of example studies where the TMF is applied, and a figure illustrating the TMF (with permissions) are also provided. These features allow for a search of the TMFs for the best fit using an algorithm that returns in order the TMFs that match the greatest number of constructs the user identifies as relevant to their project. The web tool is structured around six key action sections or pages: Plan, Select, Combine, Adapt, Use, and Assess. While the centerpiece and most complex section of the web tool is the Select section, the other sections also provide important guidance and resources for the meaningful use of the TMFs. The D&I Models web tool remains one of the most frequently used interactive tools in the field of D&I (Ford et al., 2018) with 27,046 sessions by 18,167 users between June 2021 and June 2022.

The number and use of D&I TMFs are increasing (Strifler et al., 2018). Additional knowledge and examples around how to best operationalize TMFs in D&I studies have also emerged (Damschroder, 2020; Moullin et al., 2020; Presseau et al., 2022; Snell-Rood et al., 2021). To address these new developments for D&I TMFs facilitate updates and optimize the functionality of the web tool for a wide range of users, our team conducted formal user testing on the D&I TMFs web tool to capture the needed changes and to prioritize them in alignment with the available funding to support the tool. In this paper, we explain our methodology to gather information to modify the web tool making it more user-friendly and intuitive to maximize the potential for its use in teaching, consultation, and research.

The purposes of this paper are to (1) describe the multi-step usability testing activities and their results; (2) describe the changes made to the web tool’s navigation, organization, content, and format; and (3) present recommendations for future directions for this and other interactive web tools to support capacity in D&I science.

## Methods

### Usability Testing Overview

**Fig. 1:**
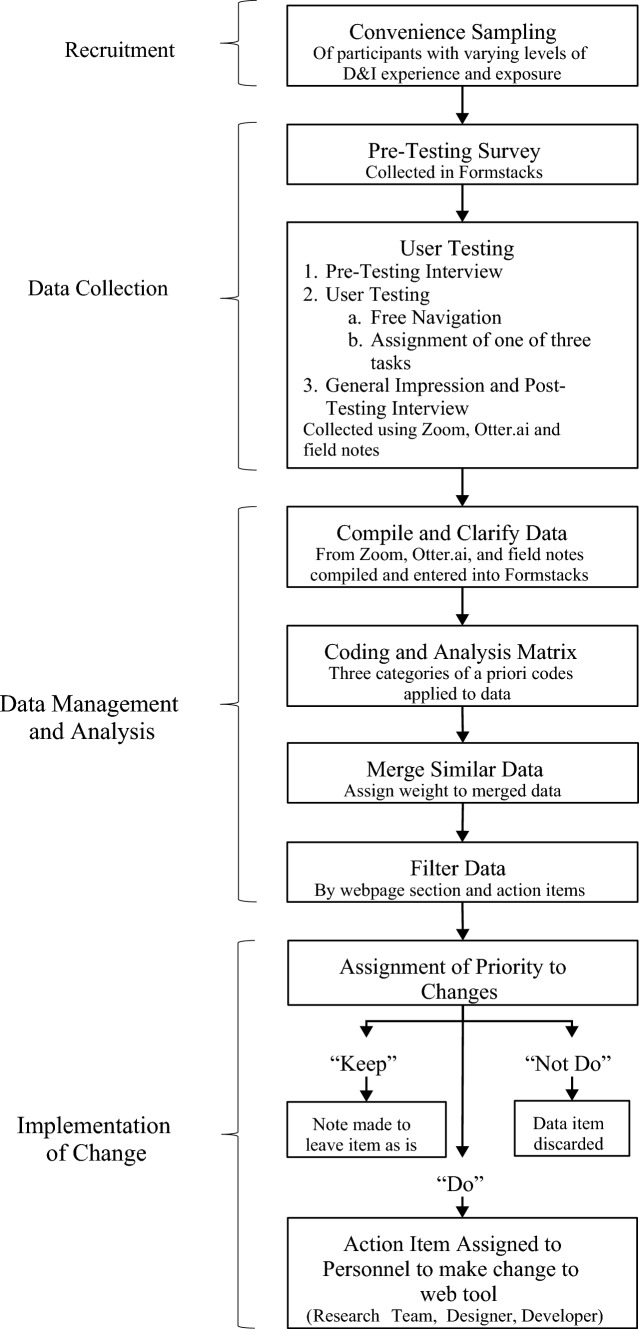
Steps to evaluate usability

We followed a usability testing protocol that involved a multi-step collection of quantitative and qualitative data. The general process of the usability testing protocol was developed for another interactive web tool using human-centered design principles and adapted for this study (Henton et al., 2017). Figure 1 summarizes processes for recruitment, usability testing, data collection, data analysis, and the implementation of changes to the web tool.

### Participant Eligibility and Recruitment

Fifteen participants were purposively selected to represent a range of disciplines and D&I expertise, all invited via a one-time email. Inclusion criteria specified having an affiliation with an academic institution (not necessarily primary appointment) and some level of experience with D&I research. For this project, participant selection was targeted at researchers as opposed to practitioners, evaluators, community members, or patients. Additional care was taken to include participants representing varying levels of research experience, D&I experience, and discipline/topical interest. We did not screen participants for or purposefully recruit them to achieve racial, ethnic, or geographic representation. Participants were not compensated.

### Data Collection Procedures

The project was a collaboration between researchers from the University of Colorado School of Medicine and Washington University at St. Louis. The research team consisted of three Masters trained research staff (RNG, BF, MP) and one PhD methodologist (SM), led by (BR) and advised by senior D&I science researchers (RT, RG, RB) who have been involved in the initial development and further expansion of the web tool. The research staff participated in training to conduct the usability testing and led the recruitment, consenting, and data collection and analysis efforts.

### Pre-test Survey

After responding to the initial invitation e-mail, participants were sent a pre-test survey to be completed before the interview. The survey asked for age, title, department of affiliation, discipline of highest degree, year received degree, and expertise in D&I (i.e., novice, advanced beginner, intermediate, advanced). The survey also asked for D&I experience in their work to date, frequency of use of D&I TMFs in their jobs (i.e., never, weekly, monthly, yearly), and the stage(s) of research the participant applied D&I TMFs (i.e., planning, implementation, adaptation, evaluation, sustainment). Finally, participants indicated which D&I TMFs they most frequently used, the resources they have used previously for selecting and using D&I TMFs, and any prior use of the D&I TMFs web tool (see Appendix A).

## Usability Testing

Usability testing sessions were conducted virtually using the Zoom platform (*Zoom Video Communications*) in Spring 2020. All participants had computer access with Zoom capabilities and consented before starting the usability testing session. Sessions were approximately 90 minutes and were conducted by four research team members in pairs (RNG, BF, SM, MP). The usability testing portion of the data collection consisted of three parts: (1) a pre-testing interview; (2) hands-on usability testing; and (3) post-testing interview. Pre-testing and post-testing interviews used a semi-structured interview guide. Pre-testing interviews included questions about expertise in and experience with D&I, experience using D&I TMFs, factors considered when choosing a D&I TMF, key challenges with selecting and using D&I TMFs, and the most common D&I TMFs they use (see Appendix B). The hands-on usability testing session used a think-aloud approach and included a 10-min free navigation session, followed by two navigation tasks and one (of two) randomly assigned “find this” tasks. This was followed by questions about general impressions within each section (see Appendix C). Post-testing interview questions inquired about overall ease or difficulty of navigation, anticipated use of the D&I TMFs web tool, intention and intended ways of using the web tool in the future, and considerations of recommending the web tool to others (see Appendix D). Research team members would assign participants one of the two “find this” tasks by alternating between the tasks. The assigned tasks asked participants either to find the Models Page or to find the construct acceptability/feasibility.

All usability testing sessions were recorded using Zoom and transcribed using Otter.ai (Otter.ai, 2016). Field notes were made on a form that followed the flow of the interview and included subsections for each page of the web tool allowing for organization and prompt data capture. Notetakers captured movement of the mouse, use of icons, links, and buttons, length of time spent per page, and directionality and order of movement amongst pages. Field notes and transcriptions were reconciled with the video recordings.

This research study received human subjects exemption approval from the Colorado Multiple Institutions Review Board.

## Data Analysis

Data from the pre-testing surveys were summarized as frequencies. Data from the usability testing sessions were analyzed and organized using a hybrid approach of deductive and adapted, rapid matrix qualitative analysis (Gale et al., 2019; Hamilton, 2020; Nevedal et al., 2021). The deductive codes used are outlined in Table 1. To reduce the vast quantity of data collected from the usability testing sessions, two members of the research team (RNG and BF) entered their field notes into an online form for each participant. The form outlined the items captured by the webpage of the web tool in the field note form. Data were condensed through matching items based on content, format, navigation, usefulness, and page. We rated the items for the plausibility of change and decided on modifications to be implemented to the web tool. Our rating system was based on assessing; (a) the capability of the web tool applications; (b) the time and funding capacity to implement the changes and; (c) the impact of the suggested change. Data from the post-test interviews were analyzed by grouped participant types based on level of expertise in D&I and degree earned. The data were also analyzed using deductive coding with a priori codes derived from the interview question. RNG added direct quotes from the transcripts to underscore the notes. A matrix categorization system, borrowing from Hamilton’s rapid qualitative analysis approach, helped the team refine the data further (Palinkas et al., 2019). Each piece of data was deductively coded by three a priori categories: main theme, user satisfaction, and by section of the web tool (see Table 1).

**Table 1:** Coding approach for the usability testing sessions

Order	Category	Code
First Code	Identifies the main theme supported	Usefulness (+ / −) Confusing Format/look Content (+ / −) Examples/Worksheets/Videos Overall design Future use Support design of D&I study Recommend to others Missing features Error
Second Code	Identifies satisfaction (when applicable)	Positive Negative Suggestion
Third Code	Identified the section of the web tool	Area of web tool where data was collected

To prioritize changes, similar comments were grouped and given a weight (count of frequency). After all the data were coded and organized, the analytic team (RNG, BF, and BR) reviewed each comment to assess the feasibility of web tool change and prioritize it based on the intent of the web tool, the weight, and the resources available. Each item was assigned an action: (1) the change would be implemented to the web tool (do); (2) an existing element of the web tool should be kept (keep); or (3) as the comment was not feasible or desirable to implement considering resources and priorities at the time (not do). The items that were marked “do” were then assigned to a team member and/or the web developer for modification and/or content creation.

## Results

### Participant Characteristics

All invited participants agreed to partake in the study and completed the full usability testing. The 15 interviewees represented diverse research and clinical groups, covering various fields utilizing D&I TMFs and expertise in D&I research (see Table 2). When asked to self-identify their D&I expertise, eight participants did so as a novice or advanced beginner, three as intermediate, and four as advanced. Most participants had a PhD (*n* = 7) and or a MD (*n* = 4). The rest of the participants had Bachelor’s, Master’s, or Occupational Therapy Doctor degrees and one participant was a doctoral student. Fields of degree included behavioral sciences and social work, biomedical sciences, instructional design, medicine, nutrition, occupational therapy, rehabilitation and participation science, public health, nursing, and health systems management and policy. The primary affiliation was distributed across a variety of health sciences departments and one participant’s primary affiliation was at a health maintenance organization. Most participants had an Assistant Professor (*n* = 5) title followed by student, research assistant, and post-doctoral research associate (*n* = 2 for each). Additional titles included, instructor, associate professor, professor, and other (*n* = 1 for each).

**Table 2:** Participant characteristics (*n* = 15) for the usability testing

	Advanced	Intermediate	Advanced Beginner/Novice	Total
Expertise in D&I (self-identified)	4	3	8	15
*Highest Degree Earned*				
PhD	3	2	2	7
MD	1	1	2	4
Bachelors			1	1
Masters			1	1
Doctoral Student			1	1
Occupational Therapy Doctorate			1	1
*Field of Degree*				
Behavioral Sciences	1	1	1	3
Biomedical Sciences			1	1
Instructional Design			1	1
Medicine	1	1	1	3
Nutrition	1			1
Occupational Therapy			1	1
Rehabilitation and Participation Science			1	1
Public Health/Nursing			1	1
Health Systems Management and Policy		1		
Social Work	1			1
Other		1	1	2
*Department Affiliation*				
Family Medicine	1		2	3
Department of Medicine	1	1	2	4
Occupational Therapy			2	2
Adult and Child Center for Outcomes Research and Delivery Science			1	1
Other			1	1
Integrated Behavioral Health, Bioinformatics	1			1
Social Work	1			1
Obstetrics and Gynecology		1		1
Public Health		1		1
*Title*				
Student			2	2
Research Assistant			2	2
Project Manager		1		1
Instructor			1	1
Postdoctoral Research Associate	1		1	2
Assistant Professor	1	2	2	5
Associate Professor	1			1
Professor	1			1

### Pre-test Survey Results

Most participants reported previous use of D&I models in their work and use of D&I models in their work on a weekly or monthly frequency. All stages of research (planning, implementation, adaptation, and evaluation) were nearly equally informed by the use of D&I models. The most used models by the participants, in order of most used to least, were Reach, Effectiveness, Adoption, Implementation, and Maintenance framework (RE-AIM) (Glasgow et al., 2019), Exploration, Preparation, Implementation, Sustainment model (EPIS) (Aarons et al., 2011), Practical, Robust Implementation and Sustainability Model (PRISM) (Feldstein & Glasgow, 2008), Consolidated Framework Implementation Research (CFIR) (Damschroder et al., 2009), Movsisyan Exploration, Preparation, Implementation, Sustainment model (Movsisyan EPIS), and Stakeholder groups. Five participants used more than one model in their research careers which included RE-AIM, PRISM, EPIS, and CFIR (See Table 3). Participants used, in order of most used to least, web tools, colleagues, and the WUSTL D&I toolkit as resources when working on D&I research projects. More than half of the participants had previously used or engaged with the D&I TMF web tool.

**Table 3:** Pre-test survey responses from participants (*n* = 15)

Expertise in D&I (self-identified)	Advanced	Intermediate	Advanced Beginner/Novice	Total
*Previous use of D&I models in work*				
Yes	4	3	3	10
No			4	4
Unknown/Missing		1	1	2
*Frequency of use of D&I models in work*				
Never			1	1
Weekly	3	2		5
Monthly	1	1	1	3
Yearly			1	1
Unknown/Missing		1	5	6
*Stages of Research Use of Models*				
Planning	3	2	2	7
Implementation	4	2	3	9
Adaptation	4	1	2	7
Evaluation	3	3	2	8
Other: Sustainment	1			1
Other	2			2
*D&I models used most frequently*				
RE-AIM	2	2	2	6
PRISM		2	1	3
EPIS	1	2	1	4
CFIR	1			
Other		1	1	2
*Past use of resources to help select D&I models*				
Yes	4		2	6
No		3	1	4
*Past resources used with selecting D&I Models*				
Research Articles	3		1	4
Web tools and web-based toolkits	2	1	2	5
Colleagues	1	1		2
*Previous use of the D&I TMF web tool*				
Yes	2	2	2	6
No	2		2	4

### Pre-test Interview Results

In the pre-testing interview, participants were asked a few semi-structured questions exploring their expertise in and experience and challenges with D&I TMFs, and which TMFs they tend to use and why.

The participants’ *expertise in and experience with D&I TMFs* varied depending on the length of their research careers, the roles or jobs they have in research, and their general exposure to D&I colleagues. Novice and advanced beginners who are also starting or had recently begun their research careers qualified their expertise based on limited exposure to D&I, by taking D&I courses and/or participating in D&I fellowships and working with D&I mentors. Novice or advanced beginners who work with D&I as research assistants, project managers, or instructors described themselves as such because they work or have extensive experience working on D&I research projects. However, they qualified their experience as they have not led these projects and did not consider themselves experts in D&I. Intermediate participants were all early-career researchers and/or new to D&I generally. They described their level of expertise as having led a few D&I research projects but not feeling like they are field experts. Advanced participants were all well-seasoned researchers who described their level of expertise as having worked in D&I research for an extensive period, usually 10-plus years, having led many D&I research projects, and having mentored younger investigators in D&I.

Participants described various *challenges with selecting D&I TMFs*. In general, participants expressed that the vast quantity of models and papers along with the plethora of jargon and a steep learning curve in the field of D&I science made selection of appropriate TMFs “overwhelming.” They reported that, instead of exploring new TMFs that may fit their projects better, they often use the same TMFs they are familiar with, were taught, or their mentors use. They also described choosing TMFs depending on the availability and complexity of available measures for any given model, tending to lean toward those with the more pragmatic measures. Overall, varying levels of expertise aside, participants expressed challenges with selecting D&I TMFs to match the needs of their specific research projects.

Various *challenges with using D&I TMFs* were described by participants. The overall use and/or adaptation of TMFs was discussed as challenging given the lack of considerations in TMFs to anticipate or adapt to local contexts and/or social determinants of health. They also described challenges with knowing how to utilize the TMFs that required assessments too difficult to implement.

### Hands-on Usability Testing Results

A total of 847 comments were identified through the matrix categorization coding system. When reviewed for similarities, comments were combined and reduced to a total of 259 unique comments. Of the 259 unique comments, 214 were positive comments about the web tool. Overall, the design and navigation of the web tool were rated positively, especially the buttons on the homepage linking to each action page of the web tool (e.g., Plan, Select, etc.) suggesting a step-by-step guide through the web tool.

So, definitely these buttons at the beginning, it was, I think, that was very helpful too and I came back several times to the homepage, so I found like it was helpful too. I knew that if I kept going back there, I could figure out where to go next. So, I think that that structure was helpful. [PhD Research Assistant]

Participants indicated that the web tool looked a bit dated due to the graphics, layout, navigation, font choice, size, and color palette.

I guess my like bottom line takeaway message would be if there’s a way to make everything more visual and less text focused, and there’s a way to err on the side of a sort of prescriptiveness and visual access and sort of reduce the amount of choice and less people go looking for it. [MD Assistant Professor]

The navigability of the web tool was assessed during the user testing interview by randomly assigning various tasks to participants. Overall, participants were able to navigate the web tool to fulfill the requirements of the task. Participants found the homepage helpful for navigation, specifically for the step-by-step design of the buttons leading to the action pages. One task asked participants to use the web tool’s search function to find a particular model. Participants were able to quickly find the search function, use the search box to enter the model’s name, and populate results. It is from this task that participants noted the need for “breadcrumbs” to help users follow their steps backward and see the mapping of location on the web tool. Participants were able to easily navigate the web tool as outlined by the task including tasks that were more self-guided and less directive.

Overall, the content was described as understandable and helpful, but the text size and wordiness generally compromised these attributes. Videos and accompanying PDFs were described as helpful and necessary but tedious, long, confusing, and lacking a flow from one PDF to another. Participants described the explanations at the top of each action page and the accompanying video (on the Plan page) as helpful for guiding the user on expectations and use of the page when needing to make the specified modifications to the models they will use for research.

Participants also described the action pages as useful for teaching users how to adapt and combine models and the limitations of doing so. Similarly, participants appreciated the readily available links to the accompanying measures for constructs, enabling users to quickly read definitions and measurement requirements. Participants found the TMF descriptions with the accompanying figures helpful for understanding the key aspects of each TMF.

Within the search action page, the search function and results were described as very helpful for narrowing a vast amount of information quickly. Participants understood how the search criteria resulted in the best match output. The search criteria/output most liked by participants was the number of citations column, noting that it quickly tells the user how “vetted” the model is by the research community.

Specific citations for the model and examples. I found that helpful… because then you know how frequently it’s used if it’s pretty common. Because some of the models are used once or twice and haven’t been vetted as much and so that that’s important to know I think. [PhD Project Manager]

Additionally, participants liked that the field of research was *not* part of the search criteria filters because it forces researchers to broaden their considerations of models for their projects. If the search criteria allowed participants to choose their field focus the output would be biased, and in not allowing it, participants said “Love this idea, it’s brilliant.” [PhD Professor].

Novice and advanced beginner researchers found the Adapt and Combine pages useful because these specifically outlined a process to adapt or combine models that the researchers otherwise would not have known how to do. However, some clarification was needed about intent, and the differences in function between those sections of the web tool. Some participants from this group anticipated more computing power from the web tool; filling fields from one PDF to another, clearly suggesting one or two models to fit the research needs, or that the Adapt or Combine pages would adapt or combine models to their projects automatically based on their inputs.

I feel like though, the good thing about the web tool is it has all the info. The bad thing about the web tool is it doesn’t actually help you come up with which one you should use. [MD Assistant Professor]

I kind of thought it wasn’t going to be just educational it was going to be actually functional. And the way that those tiles…are set up… this is an interactive web tool designed to help you develop a logic model, select our practice problem, combine models, and adapt models, it says, designed to help you combine models. Yeah. So that sort of sets [it up] …. [MD Assistant Professor]

Others from this group had different expectations and found the PDFs and the Combine and Adapt pages helpful tools walking users through all the items to consider when adapting and/or combining models.

Advanced beginners’ and Intermediate participants’ descriptions varied on the web tool’s usefulness. Some said the tool was “perhaps” helpful for thinking through processes but were unable to be explicit about how it might specifically help. Interestingly, the advanced beginners were the ones most unsure of how they would ultimately use the tool. Generally, they described it as overwhelming—especially the search function results. After entering inputs for the search function, an output of many (more than 10 or so) models was described as overwhelming to the advanced beginner. If the search output contained models that were not topically related to their research study, they found that unhelpful and not useful. They described that even after using the web tool, they would still have to dig through the suggested models reading the citations and original papers to determine which model would best fit their research needs. They also said that they would have to use this tool in tandem with expert advice from a D&I mentor. And that probably, instead of choosing a new model to experiment with, they would likely stick to using the models they were already familiar with or had already used in previous studies.

However, other advanced beginner and intermediate participants said the search function was helpful, useful, and something they would certainly utilize in their future careers. They found the construct number matches, number of times cited, and socioecological levels (see Appendix E) as helpful guides to select the models to investigate further for their research needs. They liked the search results because it narrowed the vast number of models to a handful that they could then research and select for their research needs.

The advanced D&I researcher participants found the tool overall more useful than less experienced D&I researchers. They said the web tool helps narrow vast amounts of information efficiently adding in a better search through the literature when deciding on which models to utilize for their research studies.

It advances the ability of a pretty broad range of users to get through a whole lot of complex material manageably in a manageable timeframe...As I am looking at a project and just thinking about it. I’m gonna want to go through it and use it to just think through stuff. I can see trainees, for example, also making great use of it and junior researchers too. [PhD Professor]

They suggested graphing the results of the search so one could better compare the models numerically and visually.

### Post-testing Interview Responses

After the hands-on usability testing portion was completed, participants were asked about their anticipated future use of the web tool, how supportive the tool seemed to be to their D&I TMF selecting and use needs if they would recommend the web tool to others, and who, and to provide input on what features were missing from the web tool.

Participants said they would use the D&I TMFs web tool in the future for a few key purposes. They would utilize it for planning, selecting, and adapting models. They also said they would use it to help inform and think through grant proposals, especially from a reviewer’s perspective. Participants also described using it for teaching or studying D&I. Lastly, they also said they would utilize the logic models to assist with proposal writing, construct and measures considerations, and finding citations of the original models and for use of the models. Participants also said they would recommend the web tool to colleagues, students, and the broader research community.

### Action Items from Usability Testing

Of the total of 259 comments, 142 were classified as “do” (i.e., the change would be implemented), 53 as “keep” (i.e., an existing element of the web tool should be kept), and 64 as “not do” (i.e., not feasible or desirable to implement considering resources and priorities at the time). Table 4 shows the web tool section distribution of comments across these three categories.

**Table 4:** Number of comments per section

Web tool section	Do	Keep	Not Do	Total
About Us	2	1		3
Adapt	14	10	2	26
Combine	2		1	3
Contact Us	1			1
FAQ	3		1	4
Glossary	1			1
Home	16	3	6	25
Measure	8	2	4	14
Overall	22	3	4	29
Plan	15	2	7	24
Resources	4	1	1	6
Select–Main	4		4	8
Select–Model Description	9	4	6	19
Select–Search D&I	6	4	1	11
Select–Search D&I Results	7	12	10	29
Select–View all D&I	5	4		9
Select–View Strategies	5	1		6
Submit model	2	1		3
Tutorial	13	1	2	16
Use	3	4	4	11
Grand Total	142	53	64	248

Do: the change would be implemented; keep: an existing element of the web tool should be kept’ not do: not feasible or desirable to implement considering resources and priorities at the time

For all comments that were categorized as “do”, a plan for the proposed change was made and the proposed course of action for implementing the plan was established (i.e., who should be involved and in what order in implementing the change). Considerations were made given the interview responses such as the difficulty of choosing a TMFs to make the web tool more usable for finding and sifting through the many TMFs. A sample list of some changes made to the web tool and related user feedback is listed in Table 5.

**Table 5:** Changes made to web tool

User feedback	Change Implemented to Web Tool
Remove the action page tiles from the homepage	[homepage changes, web tool moved within the website on secondary page with action page tiles]
Make it clear what the web tool does and does not do	[tutorial revamp, website landing page]
Make it clear that the site will help select a model(s) from the many out there (visually too)	[intro text or call out]
Add a blurb on each action page tile on the second page	[refine intro; copy rollover text from homepage tile to the USE intro]
Plan Page- change listing appearance (not reading the titles, only the file name)	[name the PDFs in accordion]
Re-organize PDFs	[2 columns—1 for blank; 1 for fillable—move up on page]
Update appearance to make it more visually appealing	[redesign look, use accordions, color palate change]
Make descriptions more visually appealing	[web designer]
Make the model figure bigger on the description page	[make thumbnail bigger, preview rollover]
Search D&I Page—the constructs are not alphabetical	[make in alphabetical order]
Export search results	[add xlsx; PDF]
Add “Compare” function to search results of Search D&I	[to search results at top and bottom]
Description of model: have the main paper cited at the top in big font	[add to the description of the model]

For example, the “do” comment “make it clear what the web tool does and does not do” (Table 5: Changes made to web tool based on user feedback), first, new tutorial content was developed by the research team, then the programmer added the item to the web tool. Other “do” items, such as “update appearance to make it more visually appealing,” were done by the developer and approved by the research team. The overall format, font size, style, dropdown organization, and color palette were updated to create a modern, appealing look and feel for users. Key terms were bolded that did not previously stand out to draw user attention.

The most significant change made to address the user confusion on how to access the web tool was to separate the homepage of the web tool into a main landing page and a page within that hosts the navigation buttons tool. A clearer statement of the intent and functionality of the web tool was added to the homepage to set users’ expectations. The intent of the web tool is clearly stated to be one of guiding researchers through the Planning, Selecting, Combining, Adapting, Using, and Measuring D&I research using D&I models. Instructions were also added to the main page guiding users with various levels of expertise (i.e., novice, experienced) on how to use the web tool. It also encouraged users to first visit the tutorial, glossary, or FAQ pages to learn more about how to use the web tool before moving forward and using the action pages.

To aid users with ease of navigation on the web tool, ‘breadcrumb trails’ (“a sequence of text links on the current page of a website or web-based application, usually at the top showing the page’s location within a hierarchy of content or browsing history and providing a convenient navigational tool”) (Dictionary.com, 2022) were added. Also, the navigation stage buttons were modified to provide a drop-down information feature when the buttons are rolled over by a mouse.

Introductory pages (tutorial and resources page) changed as well, some major and some minor. The tutorial page was reorganized to reflect the web tool’s step-by-step processes through Plan, Select, Combine, etc. Short introductory explainer videos now teach users about the intent of each section as well as give tips for successful use. The text in each section was also modified to streamline instruction. The resources page was arranged to reflect publications by year, making them more easily searchable. The other categories remained as the user group did not have an issue with them. Finally, links were added to the About Us page making it easier to connect with the development team and the institutions that support them.

Throughout the web tool sections, changes were enacted based on user confusion or suggestions. Each action page (Plan, Select, Combine, Adapt, Use, Assess) of the web tool was modified to include clickable drop-down features to limit the wordiness of the page and allow the user to more easily and quickly find the information they need. Worksheets for each stage and substage were redesigned to be more visually pleasing as fillable PDFs. These PDFs were reorganized to follow more logically and a thumbnail image was added. In addition, corresponding stage videos were redesigned and rerecorded to be more user-friendly, sequential, and visually pleasing.

In the Select section, a sort function was added to various variables for ease of sifting through the results. The compare function was duplicated on the top and bottom of the models list to help users locate that functionality along with a “Restore full list” button to allow users to undo their sorting of the list. The constructs found in the search models page changed from a long list to a more digestible grid format. Lastly, an Excel or CSV Format export function was added to the search results allowing users to save and share the search results.

## Discussion

The Dissemination and Implementation Models in Health web tool was created to help investigators, researchers, and others working in D&I science to sift through an overwhelming number of D&I TMFs and help them integrate these TMFs into their research projects. The changes and features mentioned by participants that could be feasibly added were added to the D&I TMF web tool. These included adding examples, definitions, and figures and having fewer words; stating the goal/intention of the web tool on the home page; and making the model search web tool more distinct from the rest of the web tool.

The ultimate goal is to build the capacity of the field of D&I and to expand its reach to the broader research and healthcare community: one key way to do this is by making D&I TMFs more widely accessible. Making successful selection and use of D&I TMFs more attainable through examples, tutorial videos, relevant publications, and guiding PDFs is key to helping novice and intermediate D&I investigators conduct high-quality projects.

The qualitative rapid, user testing methods were effective in eliciting needed feedback and responses to help the research team update and modify the web tool in a timely fashion. Testers found the site helpful in reducing the otherwise overwhelming complexity of the task of selecting and using D&I TMFs. There were several areas in which changes were recommended. The main changes made were the formatting and navigation of the web tool to make it newer, fresher, and more intuitive. In addition, some context was added or modified to better explain the web tool’s purpose, how to use the web tool and other explanations on D&I TMFs. The coloring of the pages was altered to a more professional, modern color palette and the text size was increased for better readability. In addition, the homepage was modified by removing the web tool’s navigation buttons and adding more introductory text, highlighting the portions of the web tool that teach users how to use the web tool. Changes made to emphasize the difference between the website and the embedded web tool added a second navigation page that provides explanations of web tool use to varying degrees, based on the level of user expertise.

The D&I field is currently working on developing rapid methods (Hamilton, 2020). This project used a hybrid approach combining a vetted rapid qualitative data collection method with user testing (Henton et al., 2017). This method allowed us to focus on key goals and areas of inquiry regarding the web tool and website while allowing for modest exploration of other areas of inquiry. This produced meaningful insights that could be distilled and rapidly integrated into the tool without loss of detail and essence discovered via these methods.

## Strengths and Limitations

This project had both strengths and limitations. Strengths include that the website and web tool within Dissemination-Implementation.org addresses a key need and one of the most frequently asked questions of D&I scientists: “What TMF should I use?” This resource was widely used even before this revision and update, which adds new features and improves navigation. User testing involved a diverse group of target audience users, who provided a series of actionable recommendations that led to improvements. Limitations include that the overall sample size of user testing participants (*n* = 15) was modest and systematic representative sampling methods were not used. Furthermore, information collected about the usability testing participants was limited to their professional affiliation and experience with D&I science generally, and D&I TMFs more specifically. We did not specifically recruit a diverse sample or gather information about sociodemographic characteristics such as race and ethnicity. While we believe, that due to the content area and purpose of the web tool, professional experience and D&I science and TMF expertise are the primary predictors of successful use of the web tool, in the absence of the sociodemographic data, we cannot rule out racial and ethnic disparities for usability. Future usability testing sessions should consider what additional information would be critical to gather systematically. Finally, data are not currently available to document if the revisions increase the usefulness of the web tool. As part of a new initiative, we are planning to conduct a new set of usability tests that will respond to this question.

## Future Directions

While we have made several improvements based on user testing and expanded the capacity and features of the D&I TMF web tool, it is still a work in progress and can be further improved based on experience with the new web tool. A more recent initiative completed a section on how to plan, select, combine, adapt, use, and assess TMFs and their constructs with a health equity lens (i.e., Health equity special topic). Future special topics will be added to the web tool in the coming years. Another initiative has recently begun to expand the Assess section of the web tool and provide a one-stop-shop for identifying and accessing assessment instruments aligned with the key constructs in the selected TMFs. There is a need and opportunity to evaluate the use of the web tool under different conditions to determine what level of technical assistance (if any) is required for what type of issues and for what type of users (Pawson & Tilley, 1997). Formal evaluation needs to be done to determine the use of the web tool as part of courses and training programs as well as for consultations.

## Conclusion

Overall, the web tool received many positive reviews as testers highlighted the importance of this type of tool for both early-stage and seasoned investigators in D&I science. As a result of the multi-step usability testing, there have been major improvements and expansions to the D&I TMFs web tool. To meet user needs, user testing and periodic assessments of interactive tools are recommended for other D&I capacity-building resources. To that end, iterative updating is necessary to keep the D&I Models web tool current, both in content and design. Current maintenance planning accounts for review and revision at least every five years. Future work will further expand the web tool by guiding special topics such as health equity, de-implementation, and dissemination and will attempt to expand the linkage to pragmatic measures.

## Supplementary Information

Below is the link to the electronic supplementary material.

Supplementary file1 (DOCX 22 KB)

Supplementary file2 (DOCX 21 KB)

Supplementary file3 (DOCX 25 KB)

Supplementary file4 (DOCX 19 KB)

Supplementary file5 (PDF 86 KB)

## Abbreviations

TMF: Theories, models, and frameworks
D&I: Dissemination and Implementation
