# Supplementary material for: Usability Testing of a Web Tool for Dissemination and Implementation Science Models
Source: Glob Implement Res Appl. 2024 Jun 14;4(3):296–308. doi: 10.1007/s43477-024-00125-7 (PMC11415461; doi:10.1007/s43477-024-00125-7)
Supplement: Supplementary file 1 — Supplementary file1 (DOCX 22 KB) [file 43477_2024_125_MOESM1_ESM.docx]

**Appendix A**

**Pre-Testing Survey**

Thank you for agreeing to participate in the usability testing of the Dissemination and Implementation Science Models in Health ([www.dissemination-implementation.org)](http://www.dissemination-implementation.org)) web tool.

In preparation for our interview, we would like to ask you to fill out the following brief survey.

The survey will ask few general questions about you to capture your background and expertise working in D&I and your professional affiliation. We collect these data to better understand what user characteristics are most likely to use or benefit from the web tool. We will review your responses prior to our usability testing sessions to help us understand your feedback better.

The survey should take no more than 10 minutes to complete. Answers will be kept confidential and de-identified. We will connect the answers from this survey with the data from your usability testing session.

1. What is your title?
2. With which department are you affiliated?
3. In what discipline do you hold your highest degree?
4. When did you receive this degree?
5. How much expertise do you have with dissemination and implementation science?

Would you say…

1. you are a novice (i.e., you have not engaged in any activities related to D&I research)
2. you have advanced beginner research skills in D&I (i.e., you have participated in some D&I training activities and may have contributed to a D&I proposal/project)
3. you have intermediate research skills in D&I (i.e., you have engaged in D&I-related activities in the past but have not led a proposal/project with D&I research as its main focus)
4. you have advanced research skills in D&I (i.e., you have led grant(s)/ project(s) with D&I research as its/their main focus)
5. What age range do you fall into?
6. Under 25
7. 25 - 34
8. 35 - 44
9. 45 - 54
10. 55 - 64
11. 65 - 74
12. 75 or older
13. Rather not say
14. Have you used D&I models in your work before?
15. Yes
16. No

*[If No => Go to exit statement for survey]*

1. How often do you use D&I models as part of your job?
2. Daily
3. Weekly
4. Monthly
5. Yearly
6. Never
7. Which stage or stages of your research do you typically consider applying a D&I model?
8. In the planning stage
9. During implementation
10. During adaptation
11. For evaluation
12. Other: ___________________
13. What are the D&I models you use most frequently**?**

[WRITE IN]

1. Have you used any resources in the past to help you select and/or use D&I Models?
2. Yes – [IF YES – ask to write in what resources]
3. No
4. Have you used the [www.dissemination-implementation.org](http://www.dissemination-implementation.org) web tool in the past?
5. Yes, I have checked it out
6. Yes, I have used it to inform my work
7. No
8. Not sure

Exit message: Thank you for completing this survey. We look forward to meeting you for our usability testing session.
