# Supplementary material for: Usability Testing of a Web Tool for Dissemination and Implementation Science Models
Source: Glob Implement Res Appl. 2024 Jun 14;4(3):296–308. doi: 10.1007/s43477-024-00125-7 (PMC11415461; doi:10.1007/s43477-024-00125-7)
Supplement: Supplementary file 2 — Supplementary file2 (DOCX 21 KB) [file 43477_2024_125_MOESM2_ESM.docx]

**Appendix B**

**Pre-Testing Interview Guide**

First, I would like to ask you a couple of general questions about your experience working with D&I models.

If the participant completed the pre-test survey: “Thank you for filling out the survey prior to our interview.”

Ask follow up questions on any interesting or noteworthy survey responses at this time. If not, then skip.

1. In your survey you indicated… - can you tell me more about this?

I have a couple of questions for you related to your use of D&I models:

1. What factors do you take into account when you select a D&I model from a few candidate options that may be useful for your D&I research stage (e.g., 2 or more planning D&I models, 2 or more evaluation D&I models)?
2. Please describe for me what you think is the biggest challenge when working with D&I models.

If the participant did not complete the pre-test survey:

We assembled a survey compiling some basic background information about you, your professional affiliation, and your experience with using D&I models. We collect these data to better understand what user characteristics are most likely to use or benefit from the dissemination-implementation.org tool. The survey should not take longer than 10 minutes to complete. If I resend the link to you after the testing, would you consider filling this survey out?

At this time, I would like to ask you just a couple of questions:

1. How much expertise do you have with dissemination and implementation science? [READ ANSWER OPTIONS]

Would you say…

1. you are a novice (i.e., you have not engaged in any activities related to D&I research)
2. you have advanced beginner research skills in D&I (i.e., you have participated in some D&I training activities and may have contributed to a D&I proposal/project)
3. you have intermediate research skills in D&I (i.e., you have engaged in D&I-related activities in the past but have not led a proposal/project with D&I research as its main focus)
4. you have advanced research skills in D&I (i.e., you have led grant(s)/ project(s) with D&I research as its/their main focus)
5. Have you used D&I models in your work before? If no, why not? [if no skip #7 and #8 and start usability testing]
6. What factors do you take into account when you select a D&I model from a few candidate options that may be useful for your D&I research stage (e.g., 2 or more planning D&I models, 2 or more evaluation D&I models)?
7. Please describe for me what you think is the biggest challenge when working with D&I models.

Thank you. Now we can move on to the next phase of our testing.
