# Supplementary material for: Usability Testing of a Web Tool for Dissemination and Implementation Science Models
Source: Glob Implement Res Appl. 2024 Jun 14;4(3):296–308. doi: 10.1007/s43477-024-00125-7 (PMC11415461; doi:10.1007/s43477-024-00125-7)
Supplement: Supplementary file 3 — Supplementary file3 (DOCX 25 KB) [file 43477_2024_125_MOESM3_ESM.docx]

**Appendix C**

**Usability Testing**

**Free Navigation Exercise**

10 minutes

Now, we are going to use the website and do the “think aloud” exercises I mentioned earlier. Do you have the website up in front of you? Please share your screen when you are ready. As a reminder, I am recording your activity on the screen and also what you say using the zoom recording feature.

Note to Interviewer: Any questions about the website asked at this time, defer and address after “think aloud” exercises are completed. Say “I would like you to use the tool first before I answer your questions do to this type of user testing.”

For the first exercise, I am going to give you 10 minutes to navigate the website as you so choose. I will let you know when you have 5 minutes and 1 minute left. During this exercise, feel free to visit any parts of the web tool you so wish. We would like to have you get a general sense of what this web tool can do for you and how the various functions, content, and format function for you as a user. There is a lot of information and functionality in this web tool so you might not get to everything, and that is fine. Just try to get an overall sense of what is here and how these things work.

As you are using the tool I would like you to “think-aloud” – say any and all thoughts that come to your mind as you are going through the website. This will help me understand the site from your perspective. It helps to hear why and what you are thinking as I watch you navigate the website.

There are a couple of things to keep in mind as you are using the tool:

1. You can stop at any time – just let me know if you need to stop.
2. Please give your most honest feedback about the tool.
3. The goal of having you use it is to see how well, or poorly, it works for users.

When you are ready, please share your screen and begin. You have 10 minutes.

Note to Interviewer: start timer at 10 minutes. Give a 5 and 1 minute warning. Let them navigate freely.

Note to Interviewer: if during free navigation, the user looks at a part of the web tool that is to be later covered in a task, ask the follow up questions for that task post the free navigation exercise. Skip that task later in the interview.

Free navigation follow up questions: Keep notes of interesting comments, questions the participant has and ask these in follow up.

1. How did you find the navigation and intuitiveness of the buttons on the home page?
2. You mentioned during your navigation around the website, can you tell me more about that…
3. You visited X Section during free navigation, may I ask you to go back there?

**Usability Testing: Tasks 1, 2, 3A or Tasks 1, 2, or 3B**

20 minutes

Now I would you to do a few tasks that I will walk you through.

For each task, I will read the task – please feel free to take notes on main steps. Also, I will repeat the task as needed. Feel free to ask me as you are working it through as well and I can repeat each step.

Notes to Interviewer:

- Explain each task, one at a time. Pause to let them understand it
- Observer and listen
- Probe about things that seem confusing, uncertain about
- Follow up on comments that are unclear to you
- Note if they achieved the desired outcome of the task
- Time activity from point of the end of the directions given to the point at which the participant begins undertaking the task.

Task 1: Plan Section

1. Think of a specific study that you have done or are doing and imagine that you were starting from the beginning to plan the study. Use the PLAN section of the web tool and see how you would use it to help with your planning.

Note to interviewer: Time activity from point of the end of the directions given to the point at which the participant begins undertaking the task. Let them look at the section if have not done so already

1. If they have done so: I would like you to check out the video on this page. Feel free to start it and watch as much as you think you need to understand what it is about.
   1. Would you use this on your own?
   2. Would you watch the whole thing?
   3. Would you make it longer and more detailed or shorter and less detailed?

Note to Interviewer: for the fillable pdfs, allow up to 5 minutes for the participant to fill this out. We want to see if the fillable pdf is helpful in facilitating their thought process but beyond that, such as extensive detail of their project, we are less interested.

1. If they have not done so: Would you please look at the fillable PDF for the logic model?
   1. What do you think about this resource?
   2. Can you try to fill out a few sections using the example you have been thinking about?
   3. What did you think of this pdf?
   4. Would you use this?
2. What do you think about the order of the resources in this section?
   1. Do they make sense?
   2. Would you have the example completed PDFs before or after the blank ones?

Follow up question potential:

1. If not watch video on their own: Originally, I observed you did not watch they video, why is that?
2. Was there anything particularly confusing to you?
3. Did you have trouble figuring out where to start?
4. What information are you getting from this page?
5. How would you use the information on this page?
6. Is this information useful? Why or why not?
7. Are the pages related? How?

Task 2: Search Section

I’d like to have you use the tool again. Just like with the previous task, I will read the task – please feel free to take notes on main steps. Also, I will repeat the task as needed. Feel free to ask me as you are working it through as well and I can repeat each step.

1. Please find models that meet the criteria of:
   1. Primarily an implementation focus
   2. Are appropriate at all socioecological levels
   3. And include constructs of Adoption, Acceptability/feasibility, Cost, and Fit.

Note to interviewer: Time activity from point of the end of the directions given to the point at which the participant begins undertaking the task.

Note to Interviewer: do not help them if they struggle with this. Document where they look for it. If ask for help, ask them to keep trying longer. Eventually do assist, say: “This is important for us to know that the search is hard to find. Start with going to the search function here…”

1. After letting them look at the search results and make comments about it, follow up with: What do you think of the search results?
2. Can you explain to me what the results mean to you?
   1. What do you think the “# Times Cited” means?
   2. How useful do you find this information?
   3. What do you think the “Rating” means?
   4. How useful do you find this information?
3. How would you decide which model you consider from this list?

Follow-up questions potential:

1. Overall, what is your opinion of the search function?
2. Was there anything particularly confusing to you?
3. Did you have trouble figuring out where to start?
4. What information are you getting from this page?
5. How would you use the information on this page?
6. Is this information useful? Why or why not?
7. Are the pages related? How?

Task 3A Or 3B

Note to Interviewer: Alternate between 3A and 3B with participants. Plan ahead which participants will do 3A and those that will do 3B

Task 3A: Model Page

1. Please find detailed information about the RE-AIM framework including the image of the model and ratings.

Note to interviewer: Time activity from point of the end of the directions given to the point at which the participant begins undertaking the task.

Note to Interviewer: do not help them if they struggle with this. Document where they look for it. If ask for help, ask them to keep trying longer. Eventually do assist, say: “This is important for us to know that the search is hard to find. Start with going to the search function here…”

Note to interviewer: let them review the model page for a bit and make comments. Then ask these follow up questions:

1. What do you think of this page? Do you find it useful? Is anything missing from this page?
2. What do you think of the rating and commenting section?
3. Can you try to add a rating and user comment to RE-AIM?
4. Can you identify existing measures you can use to measure constructs from the RE-AIM framework?

Follow-up questions potential:

1. Was there anything particularly confusing to you?
2. Did you have trouble figuring out where to start?
3. What information are you getting from this page?
4. How would you use the information on this page?
5. Is this information useful? Why or why not?
6. Are the pages related? How?

Task 3B: Measure Page

1. Please find detailed information about the construct ‘acceptability/feasibility.’

Note to interviewer: Time activity from point of the end of the directions given to the point at which the participant begins undertaking the task.

Note to interviewer: do not help them if they struggle with this. Document where they look for it. If ask for help, ask them to keep trying longer. Eventually do assist, say: “This is important for us to know that the search is hard to find. Start with going to the search function here…”

Note to interviewer: let them review the page for a bit and make comments. Then ask these follow up questions:

1. Can you explain to me what this information means to you?
2. Do you understand what the Elements mean?

Note to interviewer: let them find how to backtrack or return to home. Especially if they go to other sites and have difficulty getting back to d&i.org

Follow-up questions potential:

1. Was there anything particularly confusing to you?
2. Did you have trouble figuring out where to start?
3. What information are you getting from this page?
4. How would you use the information on this page?
5. Is this information useful? Why or why not?
6. Are the pages related? How?

Feedback on specific sections

And now that you completed these tasks, I would like to ask your feedback on a few specific sections of the website.

Adapt Section

1. May I ask you to go to the Adapt page?
2. How useful do you find the strategies on this page?
3. How about the example and blank worksheet?
4. How useful do you find these strategies?

Use Section

1. Now let’s visit the Use section.
2. What are your thoughts about these strategies?
3. How about the example and the worksheet?
4. How useful do you find these strategies?

Tutorial Section

1. Finally, what section of the website would you use to find guidance on how to use this web tool?
2. Can you please go to this page?
3. Can you briefly review the content of this page?
4. Do you find this useful?
   1. Too much or too little details?
   2. How about the format?
   3. Would it be useful to have a video to accompany this written text?
      1. If yes, what sections of the website require most guidance?
      2. How long video would you find optimal
5. How useful do you find these strategies?
