# Supplementary material for: Usability Testing of a Web Tool for Dissemination and Implementation Science Models
Source: Glob Implement Res Appl. 2024 Jun 14;4(3):296–308. doi: 10.1007/s43477-024-00125-7 (PMC11415461; doi:10.1007/s43477-024-00125-7)
Supplement: Supplementary file 4 — Supplementary file4 (DOCX 19 KB) [file 43477_2024_125_MOESM4_ESM.docx]

**Appendix D**

**Post-Testing Interview Guide**

20 – 25 minutes

Note to interviewer: adjust or insert task follow-up questions here. Ex: “I remember you said X earlier, would this tool make a difference in that?

Potential prompts:

- Ease of use: logical, easy, confusing, hard,
- Applicability of the results: what I need, not the information needed, not sure you to use it, useful, confusing, not sure how to interpret
- Road-blocks to adoption: takes too long, mobile device accessible, not enough expertise to use this,

Note to interviewer: if short on time, * indicates priority

Now that you have had time to use this web tool a few times, I would like to ask you a few questions. Please be frank - I didn’t design this tool so you don’t have to worry about hurting my feelings.

1. * What do you think about its overall design?
2. What was particularly easy about making your way around?
3. * What made it difficult to navigate?
4. *Could you see yourself using this site in the future? If so, for what? If not, explain.
5. Do you think this tool would make you better prepared to design an effective D&I research study? Why or why not?
6. * Would you recommend this tool to others? Who would you recommend it to? Why or why not?
7. What’s missing? What features would you like to see that would make this tool more useful for your work?
8. * Do you have any additional recommendations or comments?

Thank you. I appreciate your time.
