# Supplementary material for: Usability Testing of a Web Tool for Dissemination and Implementation Science Models
Source: Glob Implement Res Appl. 2024 Jun 14;4(3):296–308. doi: 10.1007/s43477-024-00125-7 (PMC11415461; doi:10.1007/s43477-024-00125-7)
Supplement: Supplementary file 5 — Supplementary file5 (PDF 86 KB) [file 43477_2024_125_MOESM5_ESM.pdf]

Explore D&I Models

You can search for D&I Models by entering a keyword OR by selecting from the categories below.

Model	D &/or I	Socio-Ecological Levels	Field of Origin	Times Cited
A Model for Evidence-Based Practice				
ACE Star Model of Knowledge Transformation	D>I	Individual<br>Organization<br>Community	Nursing	44
Active Implementation Framework	I-Only	Individual<br>Organization<br>Community	Education	1870
Adaptation in dissemination and implementation science	I-Only	Individual<br>Organization<br>Community<br>System	Health Disparities	39
Adherence Optimization Framework	I-Only	Individual<br>Organization<br>Community<br>System	Sports Injury Prevention	14
Advancing health disparities research within the health care system	D>I	Organization<br>Community<br>System	Health Disparities	174
Advancing Research and Clinical Practice through Close Collaboration (ARCC) Model of Evidence-Based Practice in Nursing and Healthcare	D>I	Individual<br>Organization	Nursing	1680
Availability, Responsiveness & Continuity (ARC): An Organizational & Community Intervention Model	I-Only	Organization<br>Community	Mental health	100
Behaviour Change Wheel	D>I	Individual<br>Organization<br>Community<br>System<br>Policy	Health Psychology	2430
